# Effect of Diode Laser and Sodium Hypochlorite (NaOCl) on E. faecalis in Infected Root Canals of Permanent Teeth: An In Vitro Comparative Study

**DOI:** 10.7759/cureus.117528

**Published:** 2026-10-06

**Authors:** Prashikkumar Bagade, Bikash Jyoti Borthakur, Ganesan S, Swathika B

**Affiliations:** 1 Conservative Dentistry and Endodontics, Mahatma Gandhi Postgraduate Institute of Dental Sciences, Puducherry, IND

**Keywords:** antimicrobial efficacy, diode-laser, e.faecalis, endodontic treatment, sodium hypochlorite

## Abstract

Background

Endodontic therapy requires effective disinfection of dentinal tubules to prevent treatment failure. *Enterococcus faecalis (E. faecalis)* is a resilient pathogen that penetrates dentin and resists conventional irrigants. Sodium hypochlorite (NaOCl) is widely used but has limited penetration into the tubules. Diode lasers offer deeper antimicrobial action, yet evidence at 980 nm remains limited.

Aim

To compare the antimicrobial effect of 980 nm diode laser and NaOCl on *E. faecalis* in infected root canals of permanent teeth in vitro.

Materials and methods

Fifty-five extracted permanent mandibular single-rooted premolars were standardized, sterilized, instrumented, and inoculated with *E. faecalis.* After 14 days of incubation, specimens were randomly allocated into five groups (n=11): Group A: NaOCl, Group B: saline, Group C: diode laser, Group D: diode laser with NaOCl, Group E: NaOCl with diode laser. Residual NaOCl was neutralized with 5% sodium thiosulfate. Post-treatment *E. faecalis *counts were recorded in each group and compared. Viable bacterial counts were determined by colony-forming units (CFU) analysis and expressed as log₁₀ CFU/mL. Data were analyzed using SPSS (IBM Corp., Armonk, USA). One-way analysis of variance (ANOVA) followed by Tukey's honestly significant difference (HSD) post hoc test was used to evaluate the statistical differences among the five groups.

Result

Bacterial counts differed significantly among the five groups (p<0.001). Relative to saline, NaOCl alone lowered the mean count by 20.8% (p<0.001), whereas diode laser alone did not differ significantly from saline (4.5% reduction). Both combination protocols demonstrated significantly lower post-treatment counts than either treatment alone, with diode laser followed by NaOCl achieving the lowest count (50.5% lower than saline) and differing significantly from the reverse sequence (28.5% lower than saline; p<0.001).

Conclusion

Within the limitations of this in vitro study, in which pre-treatment bacterial counts were not recorded, combining NaOCl with 980-nm diode laser irradiation resulted in lower post-treatment *E. faecalis* counts than either agent alone. Diode laser followed by NaOCl gave the lowest counts and differed significantly from the reverse sequence.

## Introduction

Endodontic therapy is fundamentally directed toward achieving three‑dimensional disinfection of the dentinal tubule walls and the root canal system. Bacterial invasion of the deeper dentin layers through necrotic pulp tissue is a principal cause of periapical inflammation [1].

Among the implicated microorganisms, *Enterococcus faecalis (E. faecalis*), a Gram-positive, facultatively anaerobic coccus, demonstrates remarkable adaptability to the harsh conditions of instrumented and medicated root canals. It exhibits resistance to mechanical instrumentation and to irrigation with antimicrobial agents, while its ability to colonize root canals, penetrate dentinal tubules, and persist despite conventional therapy has established it as a model pathogen for in vitro investigations [2].

Diode lasers are increasingly employed in endodontics for their capacity to disinfect major and accessory canals and to aid in smear layer removal [3]. Four diode laser wavelengths are commonly used: 810, 830, 940, and 980 nm. Their antimicrobial efficacy is attributed to thermal effects, with irradiation raising the temperature by approximately 7-10°C above baseline within the canal system [4].

However, evidence regarding the antimicrobial effectiveness of the 980‑nm diode laser against *E. faecalis* remains limited. Thus, the present study aims to evaluate and compare the antimicrobial efficacy of a 980 nm diode laser, 3% sodium hypochlorite (NaOCl), and their sequential combinations against *E. faecalis* in infected root canals. The null hypothesis tested was that there is no significant difference in post‑treatment *E. faecalis* counts among diode laser, NaOCl, or their sequential combinations, regardless of the order of application.

## Materials and methods

The present study was conducted in the Department of Conservative Dentistry and Endodontics of Mahatma Gandhi Postgraduate Institute of Dental Science, Puducherry, in collaboration with the Pondicherry Center for Biological Science Educational Trust (Reg. No. 34-2025 and File No. 417/MGPGIDS/IEC/2025/MDS).

Sample size calculation

The sample size was determined based on the expected difference in antimicrobial efficacy among the five experimental groups. Considering the five-group parallel experimental design and the primary outcome of bacterial reduction expressed as log₁₀ colony-forming units (CFU)/mL, an effect-size approach for comparison of multiple independent groups was used. With an assumed large effect size (f = 0.40), a significance level (α) of 0.05, and a statistical power of 80%, the minimum required sample size was estimated to be approximately 45 specimens, corresponding to nine specimens per group. To compensate for possible specimen loss during tooth preparation, sterilization, bacterial inoculation, incubation, laser irradiation, or microbiological sampling, an additional 20% was added. Therefore, the final sample size was determined as 55 teeth, with 11 specimens allocated to each of the five experimental groups.

Inclusion criteria

The study included permanent mandibular single‑rooted premolars with fully formed roots and intact root structures. Only teeth with straight or minimally curved canals were selected, ensuring standardization of canal morphology. Eligible teeth were free from caries or restorations in the root portion and had not undergone any previous endodontic treatment. Specimens were obtained from extractions performed for orthodontic or periodontal reasons, thereby minimizing pathological bias.

Exclusion criteria

Teeth with complex anatomy, such as multi‑rooted teeth or those with more than one canal, were excluded. Specimens showing calcification, obliteration, root resorption, cracks, fractures, or extraction‑related damage were also omitted. Immature teeth with open apices, previously restored or root‑filled teeth, and those with severe curvature or anatomical anomalies were excluded to avoid variability in canal preparation and microbial colonization.

Procedure

The study included fifty-five extracted permanent mandibular single-rooted premolars with fully formed roots. Specimens were rinsed thoroughly with distilled water and temporarily stored in physiological saline at 4°C. The external root surfaces were coated with a self-etching adhesive (3M Dental Products, USA) to prevent microbial contamination. Crowns were decoronated using a water-cooled diamond disc, and roots were standardized to 15 mm in length. After preparation, the apical foramina of all canals were sealed with glass ionomer cement (GC Corporation, Fuji, Japan). Working length was established using a size 10 K-file and standardized to 14 mm, 1 mm short of the apical foramen. Specimens were individually placed in sterile containers and autoclaved at 121°C and 15 psi for 20 minutes. Standardized access cavities were prepared using a sterile high-speed diamond bur under water cooling. Canals were prepared using the crown-down technique to size 35 K-file at the established working length, with sterile saline used for irrigation throughout instrumentation.

A pure colony of *E. faecalis* [microbial type culture collection and gene bank (MTCC) 2729] was cultured in nutrient broth for 24 hours at 37°C. The bacterial suspension was adjusted to 0.5 McFarland (~1 × 10⁸ CFU/mL) using sterile 0.85% saline. The standardized suspension was introduced into the prepared canals using insulin syringes, and the specimens were individually placed in sterile 2-mL centrifuge tubes and incubated for 14 days at 37°C.

Following specimen preparation and *E. faecalis* inoculation, the 55 specimens were randomly allocated in equal proportions to five experimental groups (n = 11 per group) using a computer-generated randomization sequence. Group A: NaOCl; Group B: saline; Group C: diode laser; Group D: diode laser followed by NaOCl; Group E: NaOCl followed by diode laser.

Group allocation was performed by an investigator who was not involved in the microbiological assessment. The microbiological assessor was blinded to the group allocation during sample processing, CFU enumeration, and recording of the results. Specimens were identified using coded labels to maintain blinding throughout the assessment.

Diode laser irradiation

A 980-nm diode laser, Clean Cut model DEN10B (Confident Dental Equipments, New Delhi, India), with a maximum output of 10 W, was operated at 1.5 W in pulsed mode (20 ms pulse duration, 30 Hz) using a 200-µm bare optical fiber. The fiber was positioned 1 mm short of the working length and maintained centrally within the canal without intentional wall contact. Before irradiation, the canal lumen was dried with sterile paper points; thus, irradiation was performed under dry-canal conditions, although complete desiccation of dentinal tubules was not assumed. The fiber was moved from the apical to the coronal direction at approximately 2 mm/s using controlled oscillatory motion. Four cycles of 5 s irradiation followed by 10 s cooling were performed, resulting in 20 s of cumulative irradiation and 30 J total energy delivered per specimen.

External root-surface temperature was continuously monitored using a calibrated thermocouple positioned at the middle third of the root. Temperature was recorded before, during, and after irradiation, and a maximum temperature rise of 7°C above baseline was predefined as the thermal-safety limit.

NaOCl irrigation

The canals were irrigated with 3% NaOCl using a sterile 5-mL syringe fitted with a 27-gauge side-vented needle for 60 s. The needle was positioned consistently within the canal without binding to the canal walls. At the completion of the 60 s NaOCl irrigation, negative-pressure suction was applied for 1 minute to remove the residual irrigant from the canal. The suction tip was positioned at a standardized location relative to the working length, and the same suction duration and protocol were applied to all specimens in the negative-pressure group.

Following completion of treatment, NaOCl-containing groups (Groups A, D, and E) were neutralized with sterile 5% sodium thiosulfate solution to inactivate residual NaOCl and prevent continued antimicrobial activity during microbiological sampling. The neutralizing solution was removed by aspiration followed by sterile saline flushing.

For microbiological assessment, a sterile paper point was placed within the canal and transferred into a sterile 2-mL centrifuge tube containing 1 mL sterile phosphate buffered saline (PBS). The tube was vigorously vortexed, and 0.1 mL of the resulting suspension was plated for CFU determination. The recovered viable bacterial counts were recorded and subjected to statistical analysis.

Statistical analysis

Data were analyzed using SPSS Statistics, Version 30.0 (IBM Corp., Armonk, USA). Bacterial counts were analyzed as CFU/mL and after log₁₀ transformation (log₁₀ CFU/mL) and are presented as mean ± standard deviation (SD) and median (IQR). Normality of the data within each group was assessed using the Shapiro-Wilk test. Differences among the five groups were assessed using one-way analysis of variance (ANOVA), followed by Tukey’s honestly significant difference (HSD_ post hoc test for pairwise comparisons. The percentage reduction relative to the saline control was calculated as (1 − mean of treatment ÷ mean of saline) × 100, and the log₁₀ reduction as the difference between the mean log₁₀ CFU/mL of saline and of the treatment group. A p-value of less than 0.05 was regarded as statistically significant.

## Results

Bacterial counts differed significantly among the five groups for both CFU/mL (p<0.001) (Table 1) and log₁₀ CFU/mL (p<0.001) (Table 2).

**Table 1:** Descriptive statistics of E. faecalis counts (CFU/mL) among different treatment groups *p<0.05 statistically significant; SD; standard deviation; IQR: interquartile range; NaOCl: sodium hypochlorite; E. faecalis: Enterococcus faecalis.

Group	Mean ± SD	Median (IQR)	p value	Reduction vs saline (%)
NaOCl	32582 ± 2,582	32800 (30400 – 35200)	<0.001*	20.8
Saline (control)	41118 ± 2,382	41600 (39200 – 43200)		–
Diode laser	39273 ± 3,229	39200 (36800 – 41600)		4.5
Diode laser + NaOCl	20364 ± 1,614	20800 (19200 – 21600)		50.5
NaOCl + diode laser	29400 ± 3,024	29600 (27200 – 32000)		28.5

**Table 2:** Descriptive statistics of E. faecalis counts (log₁₀ CFU/mL) among different treatment groups *p<0.05 statistically significant; SD; standard deviation; IQR: interquartile range; NaOCl: sodium hypochlorite; E. faecalis: Enterococcus faecalis.

Group	Mean ± SD	Median (IQR)	p value	Log₁₀ reduction vs saline
NaOCl	4.514 ± 0.037	4.520 (4.480 – 4.550)	<0.001*	0.100
Saline (control)	4.614 ± 0.026	4.620 (4.590 – 4.640)		–
Diode laser	4.594 ± 0.035	4.590 (4.570 – 4.620)		0.020
Diode laser + NaOCl	4.306 ± 0.035	4.320 (4.280 – 4.330)		0.307
NaOCl + diode laser	4.467 ± 0.047	4.470 (4.430 – 4.510)		0.146

Saline exhibited the highest mean colony count (41,118 ± 2,382 CFU/mL; 4.614 ± 0.026 log₁₀ CFU/mL), confirming its role as a negative control. Relative to saline, NaOCl alone reduced the mean count by 20.8% (0.100 log₁₀), whereas diode laser alone produced only a 4.5% (0.020 log₁₀) reduction. The combined regimens achieved further suppression, with diode laser followed by NaOCl yielding the lowest colony count (20,364 ± 1,614 CFU/mL; 4.306 ± 0.035 log₁₀ CFU/mL; 50.5% reduction, 0.307 log₁₀), and NaOCl followed by diode laser showing higher values (29,400 ± 3,024 CFU/mL; 4.467 ± 0.047 log₁₀ CFU/mL; 28.5% reduction, 0.146 log₁₀).

One-way ANOVA showed that bacterial counts across the treatment groups differed significantly (p<0.001). Tukey post hoc analysis revealed that NaOCl alone produced significantly lower counts than saline (mean difference −0.100 log₁₀, p<0.001) and diode laser alone (p<0.001), whereas diode laser alone did not differ significantly from saline (p=0.702). Both combination protocols demonstrated significantly lower counts than NaOCl alone (diode laser followed by NaOCl, p<0.001; NaOCl followed by diode laser, p=0.035), diode laser alone (both p<0.001) and saline (both p<0.001). Notably, diode laser followed by NaOCl had significantly lower counts than the reverse sequence (mean difference −0.161 log₁₀, p<0.001) (Table 3).

**Table 3:** Tukey HSD post hoc analysis of log₁₀ CFU/mL between groups *p<0.05 statistically significant; CI: confidence interval; NaOCl: sodium hypochlorite; HSD: honestly significant difference; CFU: colony-forming units.

Comparison	Mean difference	p value	95% CI
NaOCl vs Saline	−0.1000*	< .001	−0.1441 to −0.0559
NaOCl vs Diode laser	−0.0800*	< .001	−0.1241 to −0.0359
NaOCl vs Diode laser + NaOCl	0.2073*	< .001	0.1632 to 0.2513
NaOCl vs NaOCl + diode laser	0.0464*	.035	0.0023 to 0.0904
Saline vs Diode laser	0.0200	.702	−0.0241 to 0.0641
Saline vs Diode laser + NaOCl	0.3073*	< .001	0.2632 to 0.3513
Saline vs NaOCl + diode laser	0.1464*	< .001	0.1023 to 0.1904
Diode laser vs Diode laser + NaOCl	0.2873*	< .001	0.2432 to 0.3313
Diode laser vs NaOCl + diode laser	0.1264*	< .001	0.0823 to 0.1704
Diode laser + NaOCl vs NaOCl + diode laser	−0.1609*	< .001	−0.2050 to −0.1169

## Discussion

The morphology of root canals and dentinal tubules is an important consideration in the treatment of endodontic failure in permanent teeth. Thus, the elimination of microbial toxins and debris from the root canal is essential for the success of root canal therapy. During root canal preparation, a portion of the outer layer of the infected dentinal walls can be removed using a chemomechanical technique. The aim of the present study was to evaluate and compare the antimicrobial efficacy of a 980 nm diode laser, 3% NaOCl, and their sequential combinations against *E. faecalis* in infected root canals.

In the present study, both combination protocols lowered post-treatment *E. faecalis* counts significantly more than either agent used alone, and diode laser followed by NaOCl gave significantly lower counts than the reverse sequence. The count with NaOCl alone was significantly lower than with saline (20.8% lower), whereas the 980-nm diode laser alone did not differ significantly from saline. 

The treatment sequence may influence antibacterial outcomes because photothermal and chemical mechanisms may interact synergistically or antagonistically. Application of the diode laser prior to NaOCl irrigation could disrupt bacterial biofilms and dentinal structures, thereby enhancing penetration of the irrigant. Conversely, initial NaOCl irrigation may alter tissue properties or reduce laser absorption, potentially diminishing the photothermal effect. Evaluating both sequences provides insight into whether treatment order contributes to improved disinfection efficacy. The finding that diode laser followed by NaOCl gave lower counts than the reverse sequence is consistent with the first of these possibilities, although the mechanism was not examined directly in this study.

According to a study by Manikandan et al., *E. faecalis* produces biofilm at different pH values (7.3-12.3), and this biofilm is more susceptible to the antimicrobial effects of NaOCl than chlorhexidine [5]. Various new needle equipment and needle tip designs are becoming available to address irrigation problems in various ways, since the mode of delivery determines the efficacy and safety of irrigation. Studies indicate that diode lasers exhibit antimicrobial effects during root canal disinfection, and lasers have recently shown considerable potential in endodontics. According to Moritz et al., the root canal walls may be disinfected with an 890-nm diode laser [6]. However, in the present study, the 980 nm diode laser used alone did not differ significantly from saline, whereas its combination with 3% NaOCl was associated with the lowest CFU counts (50.5% lower than saline)

The diode laser was preferred in the present study due to its antimicrobial properties and low cost. Moritz et al. discovered a response between the ions generated by the diode laser and chemicals on the cell wall, which they attributed to the antibacterial mechanism [6]. The bacterial cell membrane was ruptured by this action, thereby damaging the cell wall protein molecules. Furthermore, the laser's primary antimicrobial effect is thermal, increasing the temperature in root canals via radiation, which disrupts bacterial cell membranes [7]. Moogi et al. stated that photoactivated disinfection with a diode laser was more effective at reducing or eliminating the bacterial load from the canals than NaOCl irrigation alone [8]. The 980-nm diode laser and 1064-nm Nd:YAG laser are expected to share similar mechanisms of action, as both operate in the near-infrared region of the electromagnetic spectrum. Mineral content of dentin, such as carbonate and phosphate, absorbs energy in these wavelength ranges, causing crystalline organisation and melting [9].

Laser energy can travel through dentinal tubules, scatter, and diffuse due to the low absorption coefficients of water and hydroxyapatite. Diode and Nd:YAG lasers are two types of laser systems with wavelengths between 980 and 1064 nm, which are near the infrared portion of the electromagnetic spectrum [10-13].

Findings of the current investigations are consistent with those of Mithra et al., Preethee et al., and Castelo-Baz. et al., who found that the diode laser, when combined with traditional chemomechanical methods, significantly reduced the number of *E. faecalis* in the root canals [14-16]. Diode laser irradiation penetrates up to 1,000 μm into dentinal tubules, compared with chemical disinfection, which penetrates only up to 300 μm, explaining its greater antimicrobial action [17,18].

Irrigant penetration has been found to be limited as the diameter of deeper dentinal tubules shrinks progressively. Because laser irradiation has built-in light scattering, local intensity augmentation, and attenuation capabilities, it can penetrate deeper into the dentinal tubules, increasing its antimicrobial activity [6]. Dai et al.'s study states that laser beams and NaOCl must be used together to completely clean the root canal [19].

Prasada et.al. found that diode laser-activated NaOCl is much more effective than ultrasonically and diode-activated chlorhexidine (CHX) and chitosan. CHX activated with ultrasonics is significantly more effective than chitosan activated with the diode laser [20]. Because diode lasers can eliminate root canal bacteria and the smear layer, they have improved the success rate of endodontic therapy. This clearly shows the advantages of laser treatments over currently used conventional irrigation methods and techniques [21]. 

One of the study's major limitations was that the study was conducted in a lab rather than a clinical environment. In addition, biofilm in clinical conditions is multidimensional, consisting of several species of microorganisms, while in our study, it was recognised as a single species containing *E. faecalis*. Furthermore, it is advised that future research examine the antimicrobial efficacy of laser beams in root canals using clinical investigations and adjustments to laser irradiation methods. Paper-point sampling was used to quantify viable intraluminal bacteria by CFU assessment; however, it may not detect microorganisms persisting within deeper dentinal tubules. Therefore, reductions in CFU counts should be interpreted as reductions in cultivable intraluminal bacteria rather than complete bacterial elimination. Also, baseline bacterial count was not recorded from individual specimens prior to treatment. As a result, the study cannot confirm within‑specimen bacterial reduction and instead reports differences in post‑treatment CFU counts across groups. This limits interpretation of the findings to between‑group comparisons rather than absolute bacterial reduction.

## Conclusions

Within the limitations of this in vitro study, specimens treated with a 980-nm diode laser combined with 3% NaOCl had significantly lower post-treatment *E. faecalis* counts than those treated with either agent alone. Laser irradiation followed by NaOCl gave the lowest count and differed significantly from the reverse sequence. Further standardized studies recording baseline counts and using mature multispecies biofilms and clinical models are needed to confirm these findings.
